# ASCL2-driven SMOC2 expression enables colorectal cancer malignant self-sufficiency via boosting TGF-β1/SMAD3-mediated EMT program

**DOI:** 10.1016/j.gendis.2026.102127

**Published:** 2026-03-05

**Authors:** Tao Jiang, Yun Liu, Yangyang Shang, Lijian Ran, Li Liu, Rongquan Wang, Jun Ye

**Affiliations:** Institute of Gastroenterology of PLA, Southwest Hospital, Army Medical University (Third Military Medical University), Chongqing 400038, China

Epithelial–mesenchymal transition (EMT) and its reverse process, mesenchymal–epithelial transition (MET), collectively termed EMT-MET plasticity, are crucial for tumor progression and metastasis.^1^ This study identifies a novel signaling axis, ASCL2/SMOC2/TGF-β1/SMAD3, as a key regulator of this plasticity in colorectal cancer (CRC). We demonstrate that the transcription factor ASCL2 (Achaete scute-like 2), a Wnt target,^2^ directly binds to the promoter of SMOC2 (SPARC-related modular calcium-binding 2), a tumor microenvironment component,^3^ and drives its transcription. ASCL2-driven SMOC2 expression subsequently activates the TGF-β1/SMAD3 signaling pathway, promoting EMT, enhancing cellular invasion, migration, proliferation, and tumor growth *in vivo*. Pharmacological inhibition of TGF-β receptors with A83-01 reversed the EMT program and associated malignant phenotypes induced by this axis. While ASCL2 expression alone was not prognostic in CRC patients, high SMOC2 expression and combined high ASCL2/SMOC2 expression were significantly associated with poorer overall and progression-free survival. Gene Set Enrichment Analysis further corroborated the link between high ASCL2/SMOC2 co-expression and enrichment of EMT and intestinal stem cell signatures. These findings establish the ASCL2/SMOC2/TGF-β1/SMAD3 axis as a critical regulator of EMT-MET plasticity in CRC, revealing a potential therapeutic target for counteracting mesenchymal traits in aggressive CRC subsets.

Our investigation began by establishing a correlation between ASCL2 and SMOC2 expression in CRC. Expression volcano plot and heatmap of differentially regulated genes in ASCL2-interfered LS174T cells (Fig. 1A and B) indicated that ASCL2 and SMOC2 transcriptional levels decreased 5.57–fold and 7.17-fold, respectively, in sh-ASCL2/LS174T cells compared to sh-Ctr/LS174T cells.^4^ Analysis of clinical datasets confirmed a positive correlation between ASCL2 and SMOC2 mRNA levels in human CRC tissues (GSE13294) (Fig. 1C), and both genes were significantly overexpressed in CRC compared to adjacent non-cancerous mucosa (GSE62321). Experimentally, shRNA-mediated knockdown of ASCL2 in HT-29 and LS174T CRC cell lines consistently reduced both ASCL2 and SMOC2 expression at the mRNA and protein levels, confirming their co-regulatory relationship (Fig. S1; Table S1, Table S2).

**Figure 1:**
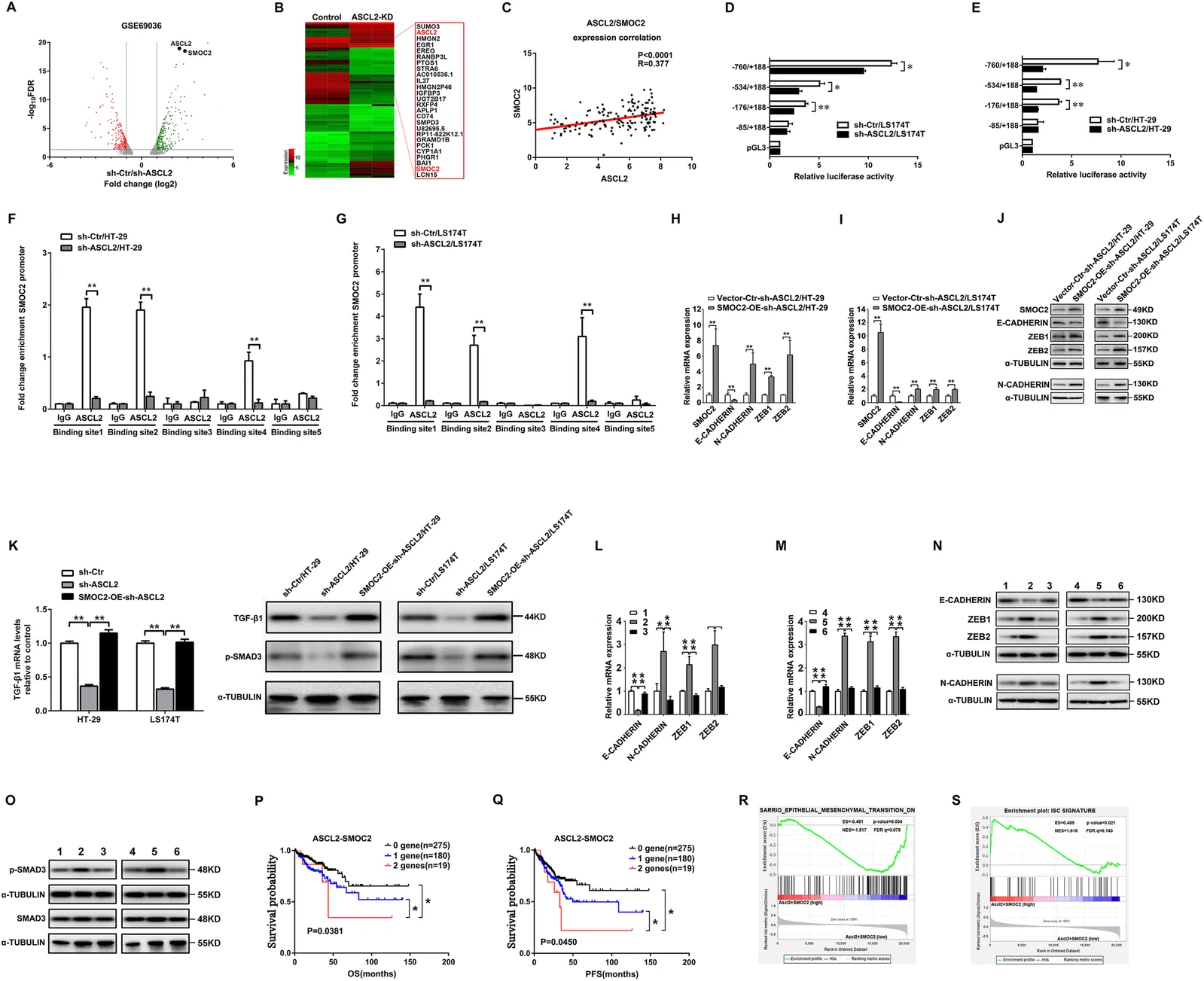
ASCL2/SMOC2 promotes colorectal cancer (CRC) malignancy via TGF-β1/SMAD3-EMT. **(A, B)** Expression volcano plot (A) and heatmap (B) of the downstream genes in sh-ASCL2/LS174T cells relative to sh-Ctr/LS174T cells from GEO profiles (GSE69036) indicated that the candidate genes enriched in sh-ASCL2/LS174T cells are enlarged in the red rectangle and both ASCL2 and SMOC2 are marked as red (B). **(C)** Correlation analysis between ASCL2 and SMOC2 mRNA expression levels in CRC tissues from GEO profiles (GSE13294). **(D, E)** The sh-ASCL2/LS174T and sh-ASCL2/HT-29 cell lines, along with their corresponding control cells, underwent transfection with SMOC2 constructs to elucidate the transcriptional regulatory regions present within the human SMOC2 promoter. The results are expressed as the mean ± standard error (SEM) derived from three independent experimental repetitions. **(F, G)** Chromatin immunoprecipitation assays revealed that ASCL2 is directly associated with the promoter of SMOC2 in both sh-ASCL2 and sh-Ctr HT-29 cells (F), as well as in sh-ASCL2 and sh-Ctr LS174T cells (G). **(H**–**J)** In relation to sh-NC controls, SMOC2-OE-sh-ASCL2/HT-29 and SMOC2-OE-sh-ASCL2/LS174T cells showed an downregulation of E-CADHERIN (epithelial marker) and a upregulation of N-CADHERIN/ZEB1/ZEB2 (mesenchymal markers) at both mRNA and protein levels. **(K)** The TGF-β1 mRNA and protein levels, and p-SMAD3 protein were observed in the homogenized tissues derived from tumor xenografts originating from sh-ASCL2/HT-29, sh-ASCL2/LS174T, and SMOC2-OE-sh-ASCL2 cell lines. **(L**–**N)** A comparative analysis was conducted to examine the mRNA and protein expression levels of E-CADHERIN, N-CADHERIN, ZEB1, and ZEB2 in various cell lines, specifically focusing on sh-ASCL2/LS174T and sh-ASCL2/HT-29 cells, as well as SMOC2-overexpressing sh-ASCL2 cells and those treated with A83-01. **(O)** A83-01 treatment in SMOC2-OE-sh-ASCL2 cells reduced the p-SMAD3 protein levels, did not affect the SMAD3 mRNA and protein levels (1: sh-ASCL2/HT-29, 2: SMOC2-OE-sh-ASCL2/HT-29, 3: SMOC2-OE-sh-ASCL2/HT-29+A83-01, 4: sh-ASCL2/LS174T, 5: SMOC2-OE-sh-ASCL2/LS174T, and 6: SMOC2-OE-sh-ASCL2/LS174T+A83-01). **(P, Q)** Kaplan–Meier survival analysis of combined ASCL2 and SMOC2 mRNA levels in cancerous tissues from CRC patients for OS and PFS. The three subgroups are defined as follows: “0 gene” indicates low expression of both ASCL2 and SMOC2, “1 gene” denotes high expression of either ASCL2 or SMOC2 (but not both), and “2 genes” represents high expression of both ASCL2 and SMOC2. **(R,S)** GSEA of EMT (R) and ISC **(S)** signature genes between CRC patients with high ASCL2 and SMOC2 mRNAs versus low ASCL2 and SMOC2 mRNAs. (∗*p* < 0.05, ∗∗*p* < 0.01).

We next sought to determine if this regulation was direct. *In silico* analysis of the SMOC2 promoter identified clustered potential ASCL2 binding sites (E-Boxes) (Fig. S2 and S7). Luciferase reporter assays demonstrated that ASCL2 knockdown significantly reduced the activity of various SMOC2 promoter constructs, pinpointing critical regulatory regions between −760 and −85 bp (Fig. 1D and E; Table S1). Chromatin immunoprecipitation assays provided direct evidence, showing specific enrichment of ASCL2 at three predicted binding sites (BS1, BS2, BS4) within the SMOC2 promoter in control cells, which was markedly diminished in ASCL2-knockdown cells (Fig. 1F and G). These findings unequivocally establish SMOC2 as a direct transcriptional target of ASCL2 in CRC.

Functionally, ASCL2 knockdown induced a MET-like state, characterized by increased E-CADHERIN (epithelial marker) and decreased N-CADHERIN, ZEB1, and ZEB2 (mesenchymal markers) expression (Fig. S3; Table S1 and S2). This was accompanied by significantly impaired cell migration, invasion, and proliferation *in vitro* (Fig. S3). Crucially, enforced expression of SMOC2 in these ASCL2-deficient cells effectively reversed this MET phenotype (Fig. 1H–J), restoring the mesenchymal marker profile and rescuing the impaired migratory, invasive, and proliferative capacities (Fig. S3). *In vivo*, ASCL2 knockdown suppressed tumor growth in mouse xenograft models, while SMOC2 overexpression in the knockdown cells restored tumorigenicity, as evidenced by increased tumor volume and weight (Fig. S4). This demonstrates that ASCL2-induced EMT and associated malignant behaviors are mediated primarily through its downstream target, SMOC2.

Mechanistically, we linked this axis to the well-established EMT-inducing TGF-β pathway. Analysis of tumor xenografts revealed that ASCL2 knockdown led to decreased TGF-β1 mRNA and protein levels, as well as reduced levels of phosphorylated SMAD3 (p-SMAD3), the active form of the transcription factor (Fig. 1K; Table S1 and S2). Total SMAD3 levels remained unchanged (Fig. S8). Importantly, SMOC2 overexpression in the ASCL2-knockdown background restored TGF-β1 expression and p-SMAD3 levels (Fig. 1K). This indicates that ASCL2-driven SMOC2 expression acts as an upstream activator of the TGF-β1/SMAD3 signaling cascade in CRC.

To assess the therapeutic potential of targeting this axis, we employed the TGF-β receptor inhibitor A83-01. Treatment with A83-01 in SMOC2-overexpressing, ASCL2-knockdown cells effectively reversed the SMOC2-induced EMT program. This was marked by a shift back towards an epithelial state (upregulated E-CADHERIN, downregulated N-CADHERIN, ZEB1, ZEB2) (Fig. 1L–N) and a significant reduction in migration, invasion, and proliferation *in vitro* (Fig. S5). Furthermore, A83-01 treatment markedly inhibited the *in vivo* tumor growth of these cells in mouse xenografts (Fig. S5). The inhibitor specifically reduced p-SMAD3 protein levels without affecting total SMAD3, confirming its on-target effect (Fig. 1O). This confirms that the pro-EMT effects of the ASCL2/SMOC2 axis are dependent on TGF-β1/SMAD3 signaling and are pharmacologically reversible.

The clinical relevance of our findings was evaluated using data from The Cancer Genome Atlas (TCGA) cohort of 474 CRC patients. Kaplan–Meier survival analysis revealed that high SMOC2 expression was significantly associated with worse overall survival (OS) and progression-free survival (PFS). While aberrant expression of ASCL2 has been linked to tumor metastasis and patient prognosis in osteosarcoma, breast cancer, gastric carcinoma, glioma, and lung squamous cell carcinoma,^5^ it is not related to CRC patient survival (Fig. S6).

ASCL2 expression alone did not show a significant correlation with patient survival. However, when patients were stratified based on combined abnormal expression of both ASCL2 and SMOC2, the group with high co-expression exhibited significantly poorer OS and PFS compared to patients with no or only one abnormally expressed gene (Fig. 1P and Q). Cox regression analysis further identified SMOC2 as an independent prognostic factor for OS and PFS (Fig. S6). Additionally, a correlation was found between SMOC2 levels and PFS, evidenced by a hazard ratio (HR) of 1.452, a 95% CI of 1.002–2.104, and a *p*-value of 0.049 (Fig. S9). Gene Set Enrichment Analysis (GSEA) of the TCGA data provided a mechanistic rationale for these clinical observations, showing that tumors with low ASCL2/SMOC2 co-expression were enriched for gene sets related to EMT downregulation, whereas those with high co-expression were enriched for intestinal stem cell (ISC) signature genes (Fig. 1R and S).

In conclusion, this study delineates a comprehensive signaling pathway—the ASCL2/SMOC2/TGF-β1/SMAD3 axis—that critically regulates EMT-MET plasticity in CRC. ASCL2 directly transcriptionally activates SMOC2, which in turn potentiates TGF-β1/SMAD3 signaling to drive EMT, stemness, and malignant progression. The findings clarify that while ASCL2 is a potent driver of this program experimentally, SMOC2 expression or the combined ASCL2/SMOC2 signature holds greater prognostic value in patients, potentially due to its role as the key effector amplifying the signal. The reversal of this axis using A83-01 highlights its therapeutic vulnerability. Targeting this newly identified axis, particularly in CRC patients exhibiting high ASCL2 and SMOC2 co-expression, represents a promising strategy to suppress EMT and mitigate tumor aggressiveness.

## CRediT authorship contribution statement

**Tao Jiang:** Validation. **Yun Liu:** Validation. **Yangyang Shang:** Validation, Data curation. **Lijian Ran:** Software, Methodology. **Li Liu:** Software, Formal analysis. **Rongquan Wang:** Writing – original draft, Funding acquisition, Conceptualization. **Jun Ye:** Writing – review & editing, Supervision, Funding acquisition, Conceptualization.

## Ethics declaration

All experimental protocols involving animals were formally approved by the Animal Research Ethics Committee at Army Medical University. The research was conducted under the oversight of the Institutional Animal Care and Use Committee (IACUC), ensuring adherence to the ethical principles and operational guidelines specified in the Guidelines for the Care and Use of Laboratory Animals.

## Funding

This study was supported by the 10.13039/501100001809National Natural Science Foundation of China (No. 81572903) and the 10.13039/501100005230Natural Science Foundation of Chongqing (No. cstc2020jcyj-msxmX1094).

## Conflict of interests

The authors report no potential conflict of interests.

## Figures and Tables

**Figure S1:**
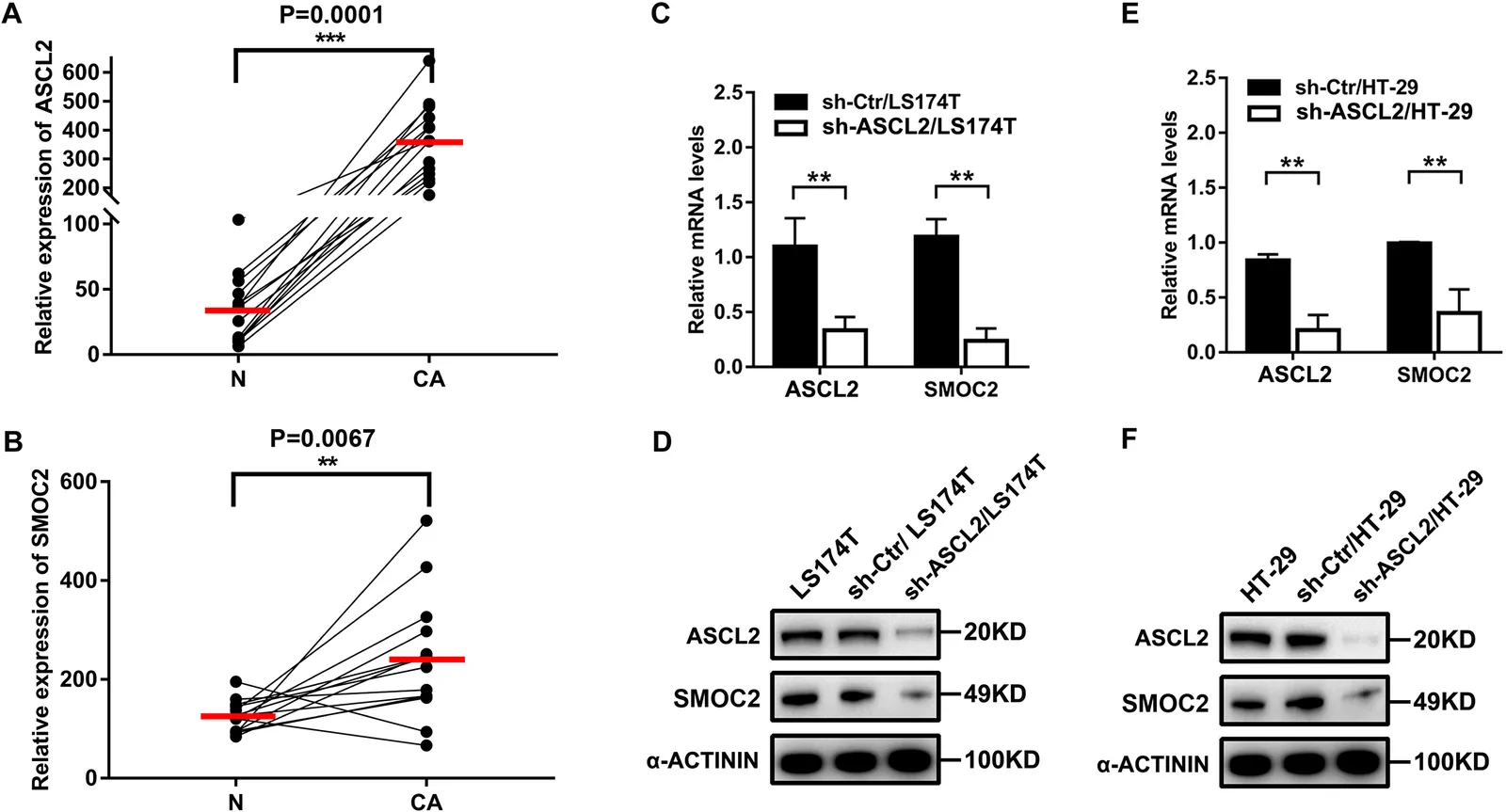
ASCL2 expression in colorectal cancer (CRC) tissues and cell lines correlates with SMOC2 expression. **(A, B)** The expression levels of ASCL2 and SMOC2 mRNA were evaluated in human CRC tissues as well as in matched pericancerous mucosal samples sourced from the Gene Expression Omnibus (GEO) dataset (GSE62321). **(C–F)** The mRNA and protein expression levels of ASCL2 and SMOC2 in sh-ASCL2-transfected HT29 and LS174T cell lines, alongside their corresponding control cells (∗*P* < 0.05, ∗∗*P* < 0.01).

**Figure S2:**
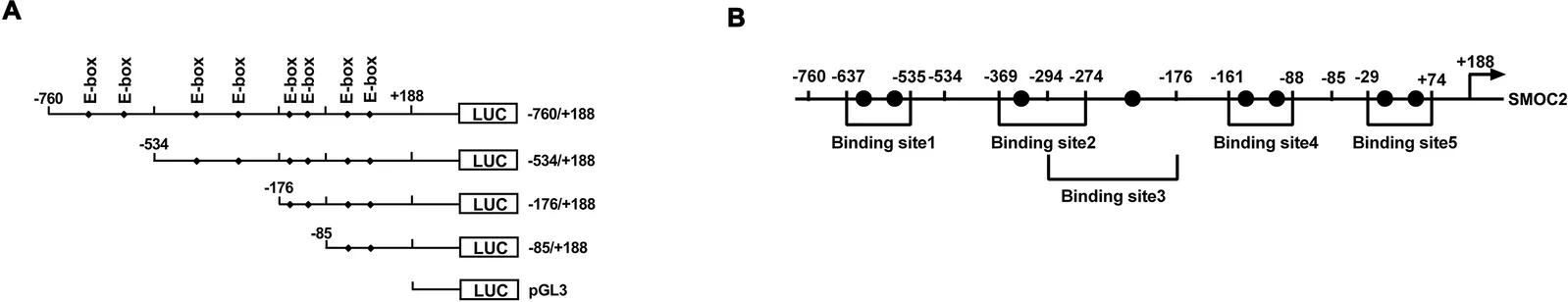
The ASCL2 gene exerts transcriptional regulation on the SMOC2 gene. **(A)** A schematic representation illustrating four distinct luciferase constructs derived from the human SMOC2 promoter. **(B)** A diagrammatic illustration of the human SMOC2 promoter is presented. Five distinct regions within the SMOC2 promoter, identified as potential ASCL2 binding sites (labeled 1–5), were evaluated.

**Figure S3:**
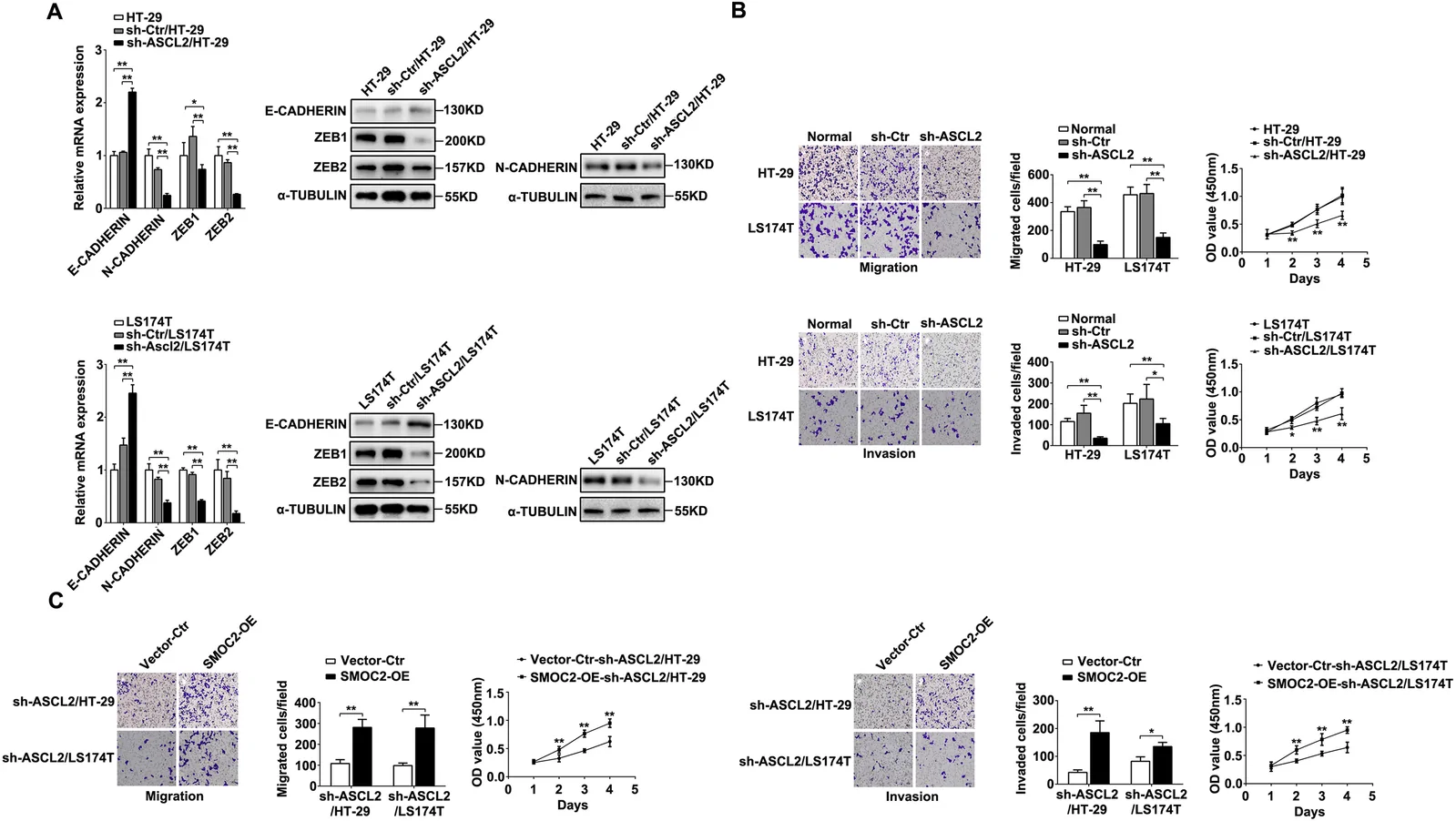
The knockdown of ASCL2 induces a MET-like state in CRC cells, an effect that is counteracted by the overexpression of SMOC2. **(A)** Relative to sh-NC controls, sh-ASCL2/HT-29 and sh-ASCL2/LS174T cells demonstrated an increased expression of E-CADHERIN, an epithelial marker, alongside a decreased expression of N-CADHERIN, ZEB1, and ZEB2, which are mesenchymal markers, both at the mRNA and protein levels. **(B)** A reduction in migration, invasion, and proliferation was observed in sh-ASCL2/HT-29 and sh-ASCL2/LS174T cells when compared to their respective control cells (Scale bar = 200 μm). **(C)** The overexpression of SMOC2 in sh-ASCL2/HT-29 and sh-ASCL2/LS174T cells (designated as SMOC2-OE-sh-ASCL2) significantly enhanced their migratory, invasive, and proliferative abilities (Scale bar = 200 μm) (∗*P* < 0.05, ∗∗*P* < 0.01).

**Figure S4:**
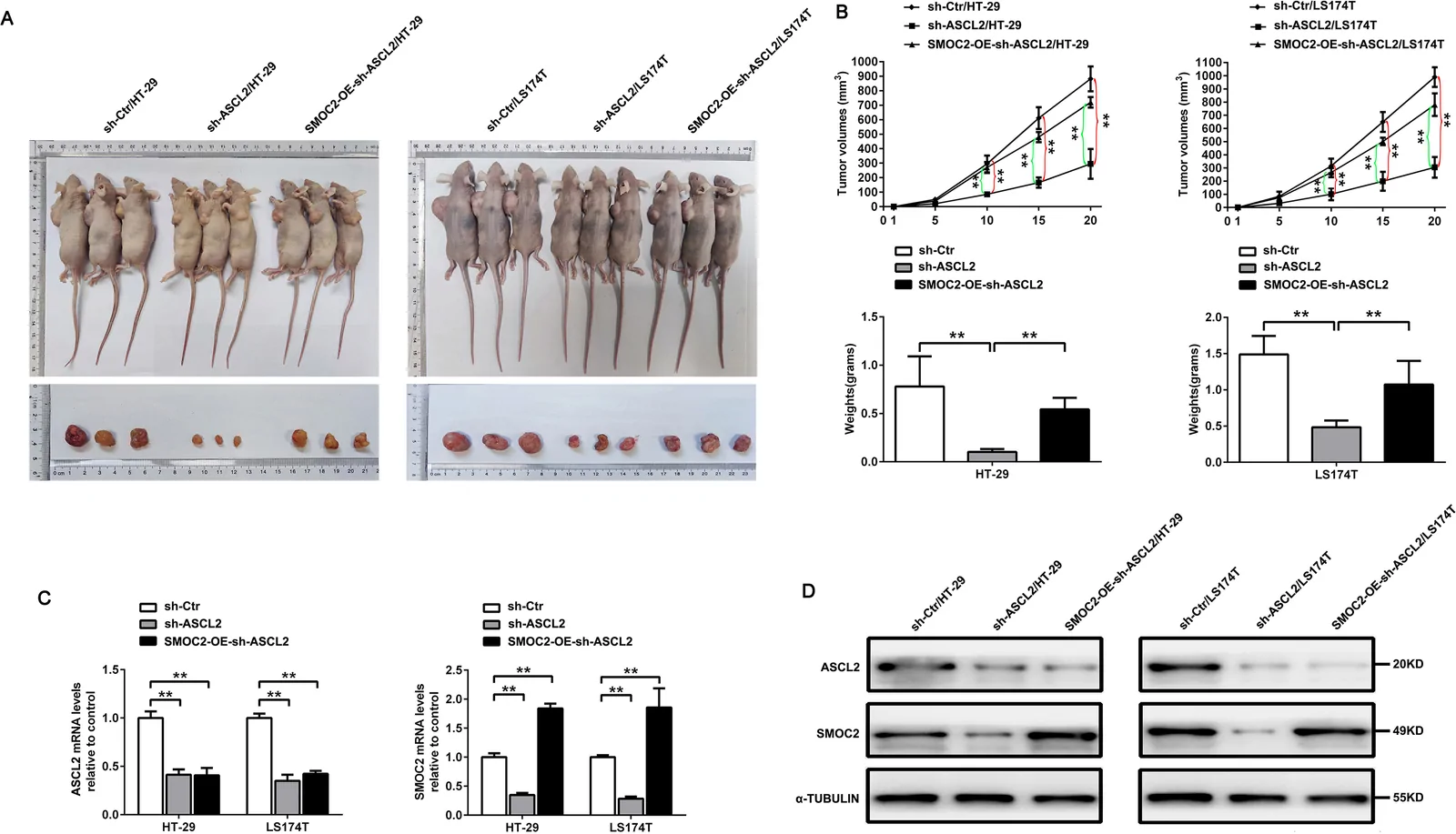
Mouse xenografts assay and SMOC2 overexpression in ASCL2 interfered CRC cells reverses TGF-β1/SMAD3 signaling which is inhibited by ASCL2 interference. **(A, B)** For mouse xenografts assay, the grafts in the nude mice were documented by photography, and their tumor size and weight were measured. **(C, D)** The ASCL2 and SMOC2 mRNA (C) and protein (D).

**Figure S5:**
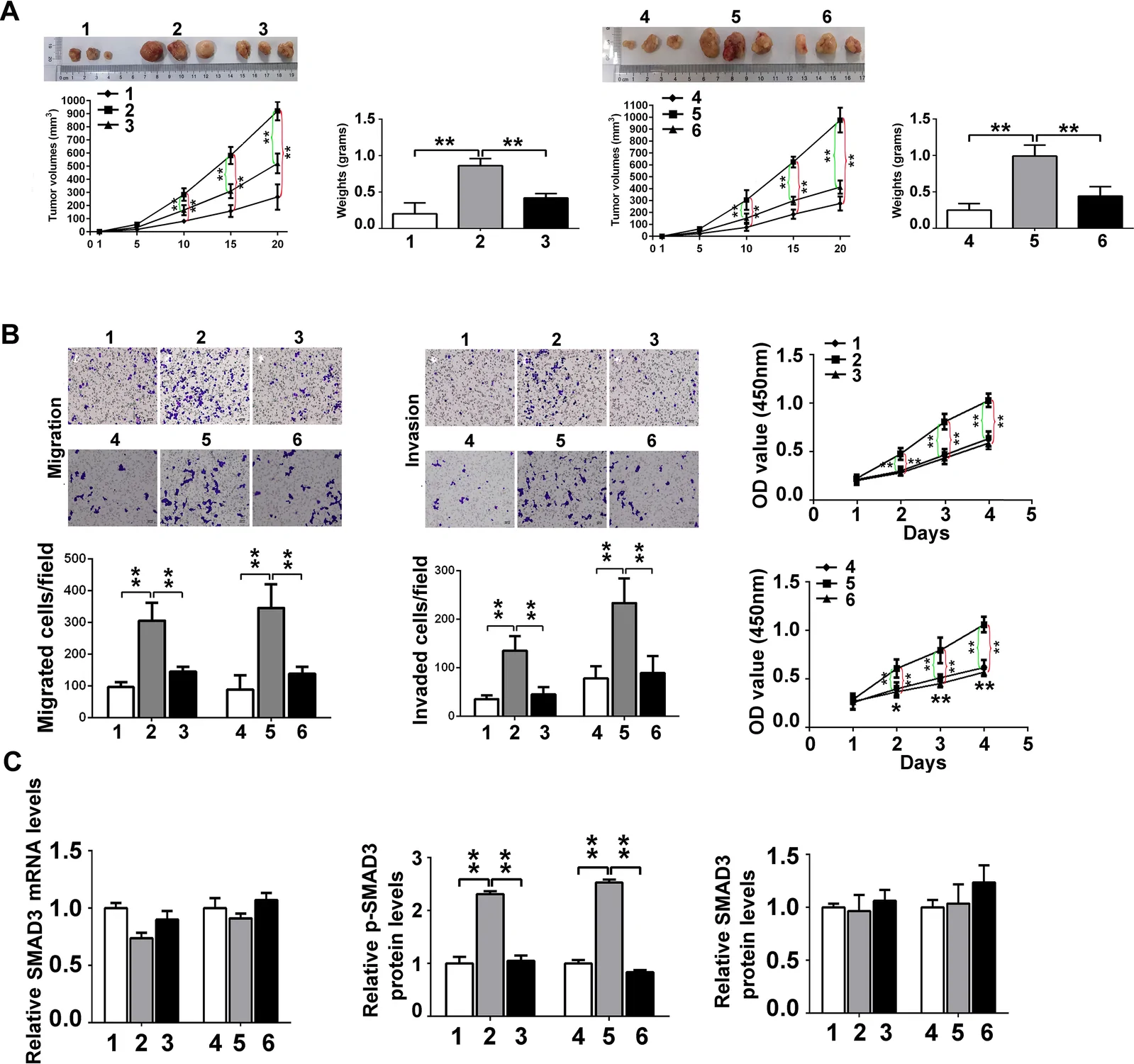
The TGFβ inhibitor A83-01 (1 μM) reverses the EMT program induced by SMOC2-enforced expression in sh-ASCL2/HT-29 and sh-ASCL2/LS174T cells. **(A)** The grafts were photographed, their volumes and weights were measured. **(B)** Comparison of their migration, invasion and proliferation. Scale bar = 200 μm. **(C)** A83-01 treatment in SMOC2-OE-sh-ASCL2 cells reduced the p-SMAD3 protein levels, did not affect the SMAD3 mRNA and protein levels (∗*P* < 0.05, ∗∗*P* < 0.01) (1: sh-ASCL2/HT-29, 2: SMOC2-OE-sh-ASCL2/HT-29, 3: SMOC2-OE-sh-ASCL2/HT-29+A83-01, 4: sh-ASCL2/LS174T, 5: SMOC2-OE-sh-ASCL2/LS174T, and 6: SMOC2-OE-sh-ASCL2/LS174T + A83-01).

**Figure S6:**
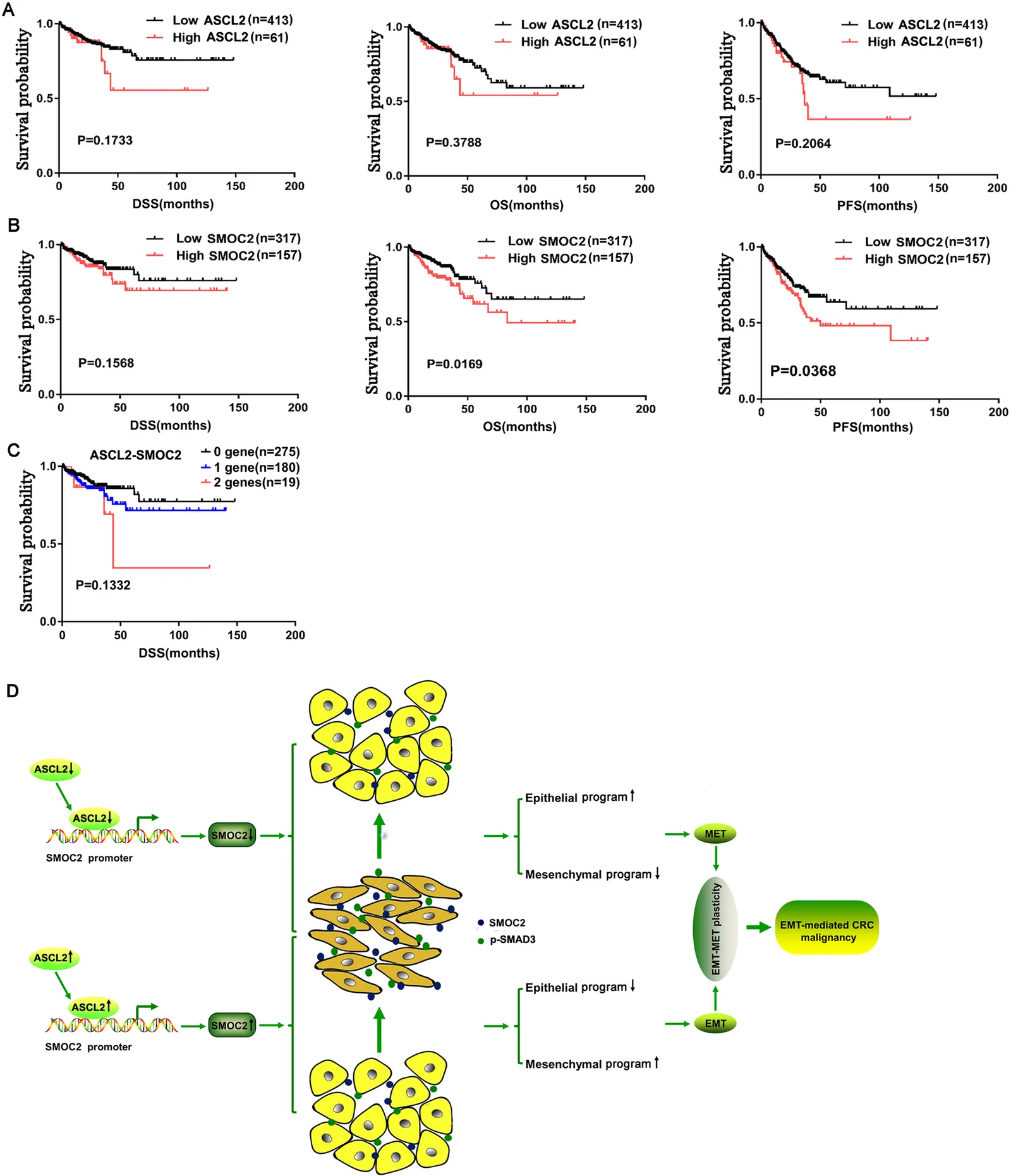
Kaplan–Meier survival analysis, GSEA of EMT and ISC signature genes, and our proposed hypothesis. **(A**–**C)** Kaplan–Meier survival analysis of ASCL2 (A), SMOC2 (B) and combined ASCL2 and SMOC2 mRNA expression patterns in CRC patients: patients were stratified into 3 subgroups based on the number of genes with high expression—0 gene (low expression of both ASCL2 and SMOC2), 1 gene (high expression of either ASCL2 or SMOC2 alone), and 2 genes (high expression of both ASCL2 and SMOC2), with DSS as the survival endpoint (C). **(D)** Our proposed hypothesis.

**Figure:**
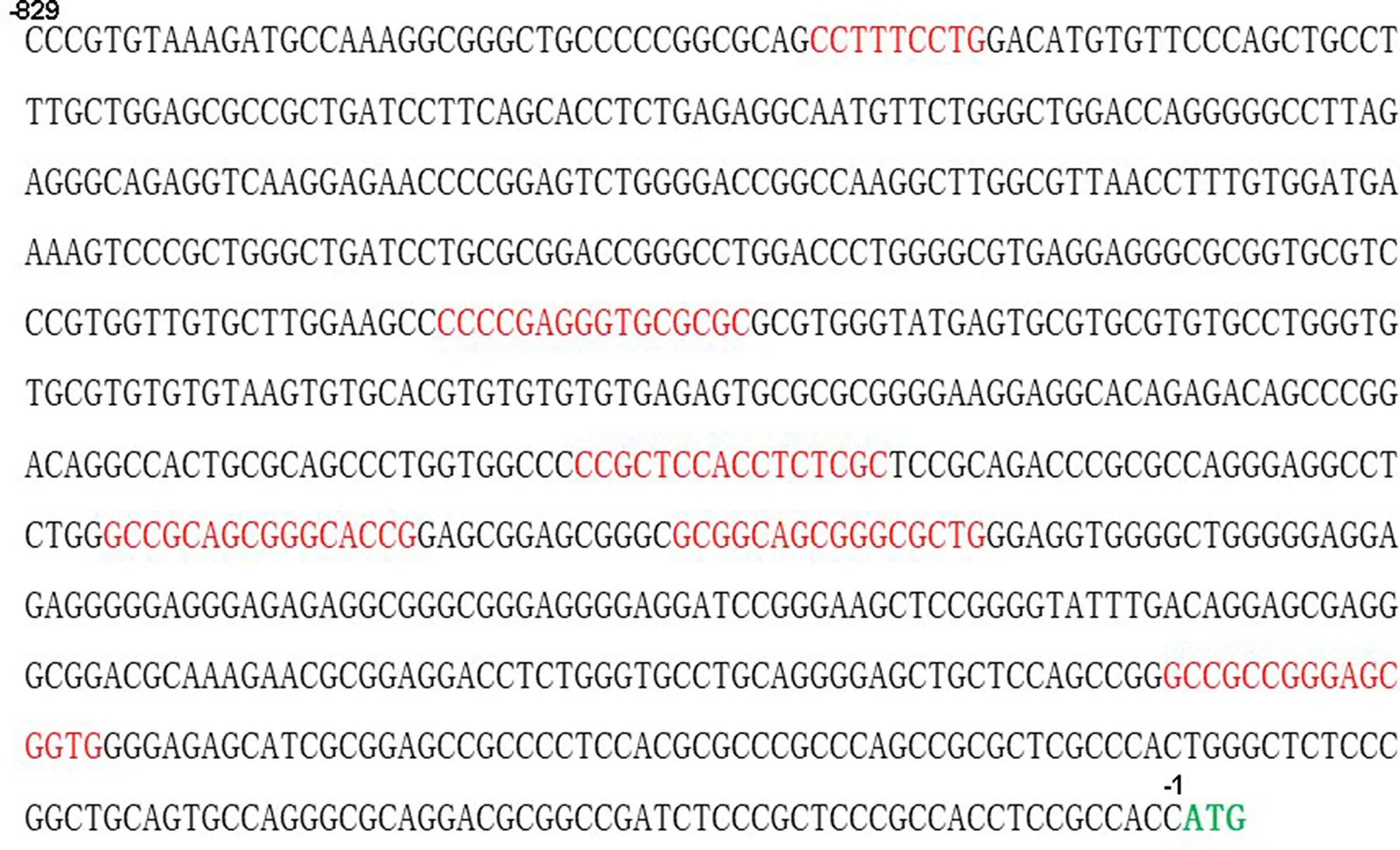
Figure S7The nucleotide sequence of the SMOC2 promoter, along with the prospective ASCL2 binding sites (E-Boxes) located within the promoter region spanning from −829 to −1, has been identified. The potential E-Box sites were marked as red.

**Figure:**
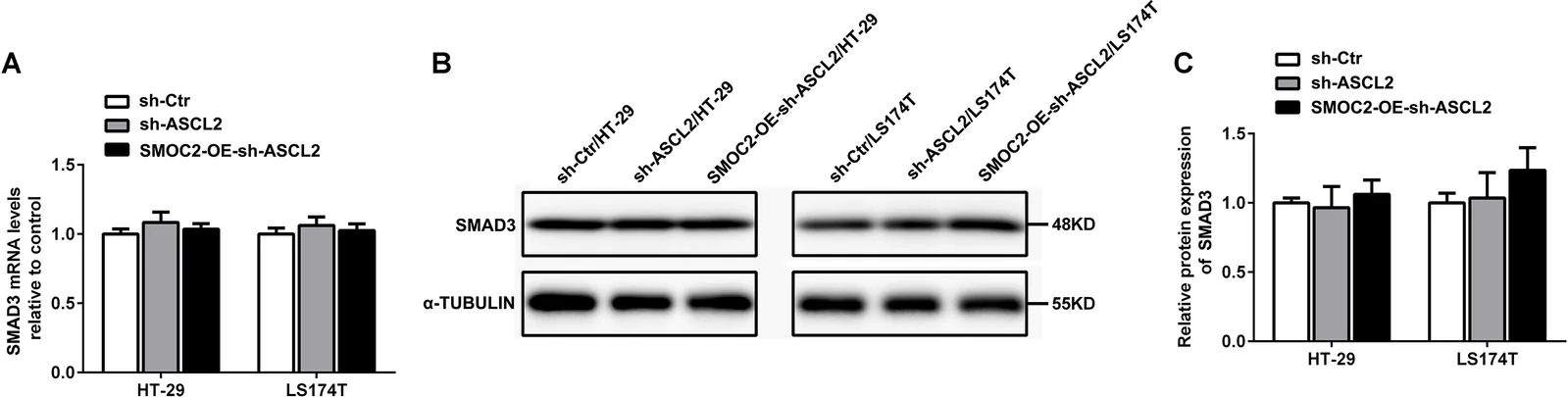
Figure S8SMAD3 mRNA (A) and protein expression (B, C) is unaffected after ASCL2 interference or their further SOMC2 enforced expression.

**Figure:**
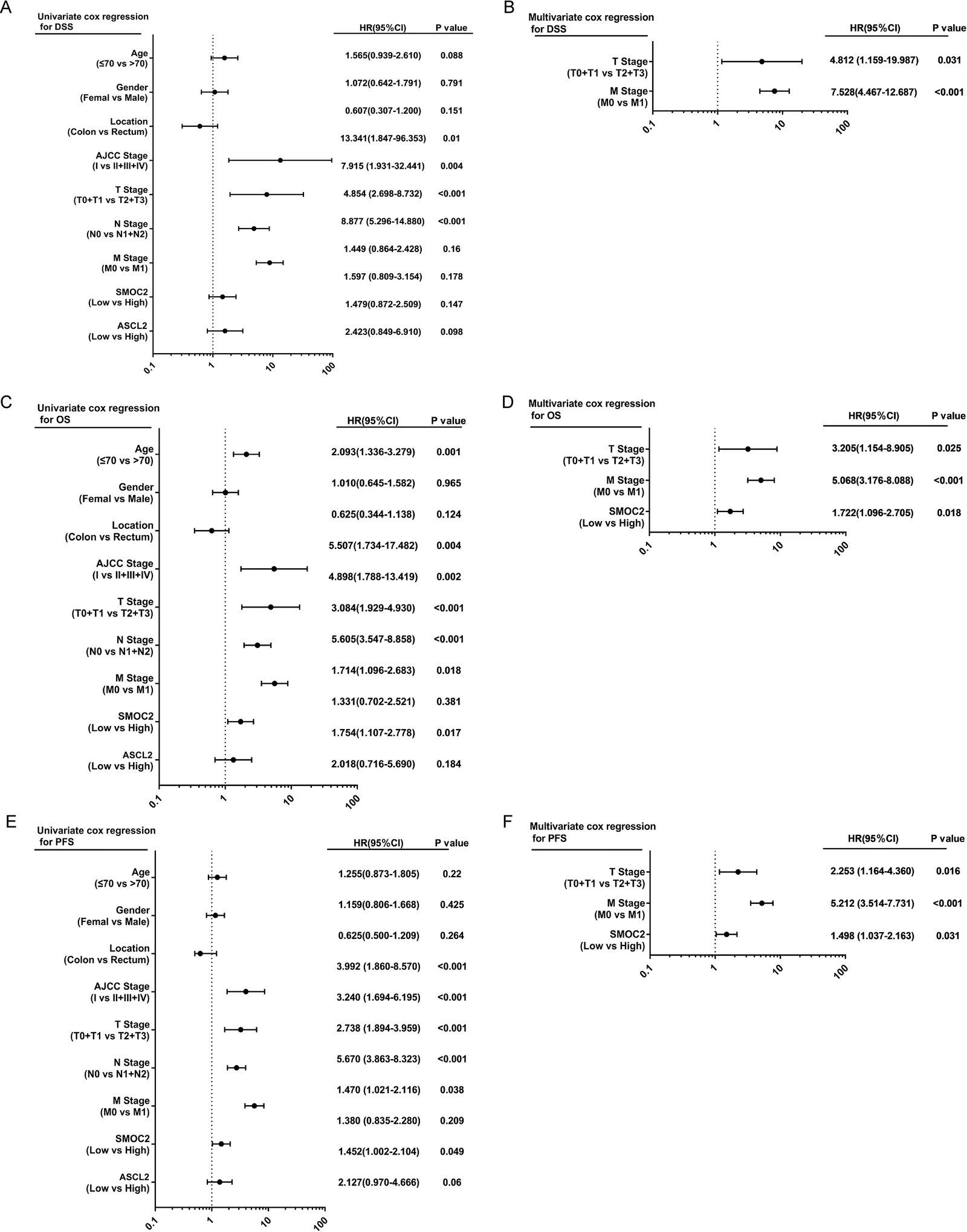
Figure S9Univariate and multivariate analysis of ASCL2, SMOC2, and combined abnormal ASCL2 and SMOC2 expression status with DSS (A, B), OS (C, D), and DFS (E, F) in CRC patients.
