# Supplementary material for: ASCL2-driven SMOC2 expression enables colorectal cancer malignant self-sufficiency via boosting TGF-β1/SMAD3-mediated EMT program
Source: Genes Dis. 2026 Mar 5;14(3):102127. doi: 10.1016/j.gendis.2026.102127 (PMC13718859; doi:10.1016/j.gendis.2026.102127)
Supplement: Supplementary file 1 [file mmc1.docx]

**Materials and methods**

***Cell culture***

The HT-29 and LS174T colon adenocarcinoma cell lines were acquired from the Typical Culture Preservation Committee of the Cell Bank located in Shanghai, China. Both of them have relatively high ASCL2 expression levels and were chosen to knockdown their ASCL2 expression. ASCL2-targeting and control short hairpin RNAs (shRNAs) were purchased from Wuhan Genesil Biotechnology Co., Ltd. Stable cell lines, designated as sh-Ctr/HT-29, sh-ASCL2/HT-29, sh-Ctr/LS174T, and sh-ASCL2/LS174T, were constructed in accordance with the protocol provided by the manufacturer. All cells were cultured in McCoy’s 5A medium supplemented with 10% fetal bovine serum (FBS) at 37°C with 5% CO₂.

***SMOC2 over-expression assay***

To overexpress SMOC2，the pEX-5 plasmid (pGCMV/EGFP/MCS/Neo) (GenePharma Co. Ltd, Shanghai, China) was inserted with the synthetic SMOC2 sequence in its multiple cloning sites (MCS) and used for SMOC2 over-expression. The sh-ASCL2/HT-29 and sh-ASCL2/LS174T cell lines were subjected to transfection with the pEX-5 vector, which harbors the SMOC2 gene, following the guidelines established by the manufacturer. In order to develop stable cell lines exhibiting overexpression of SMOC2, the transfected cells were subjected to selective pressure using G418 at a concentration of 800μg/ml, maintained over a duration of two weeks. The resultant cell populations were subsequently referred to as SMOC2-OE/sh-ASCL2/HT-29 and SMOC2-OE/sh-ASCL2/LS174T, respectively.

***qPCR and western blotting assays***

Total RNA was extracted from all cell lines employed in the study utilizing an RNA Isolation Kit (MilliporeSigma; Merck KGaA), in accordance with the manufacturer's established protocol. The integrity of the isolated RNA was evaluated by measuring the A260/A280 absorbance ratio, with values between 1.8 and 2.0 deemed indicative of high RNA purity; these assessments were carried out using a NanoDrop 2000 spectrophotometer (Thermo Fisher Scientific, Inc.). Variations in the expression levels of the target indicators were analyzed through quantitative real-time polymerase chain reaction (qPCR) technology. The sequences of the qPCR primers specific to the target genes, along with the respective annealing temperatures for each primer pair, are detailed in Supplementary Table 1. α-TUBULIN was used as the internal reference gene for normalization. The subsequent evaluation of the qPCR experimental outcomes was performed using Bio-Rad CFX Manager software (version 3.1; Bio-Rad Laboratories, Inc.).

Total proteins were extracted from all cell lines following the instructions provided with the Protein Extraction Kit (Beyotime Biotechnology Co., Ltd.). The primary antibodies, which are employed for the precise identification of specific antigens, along with the secondary antibodies utilized in the Western blotting procedure, along with their respective sources and catalog numbers are listed in Supplementary Table 2.

***MTT assay***

For cell lines requiring proliferation detection, cells were seeded into 96-well plates at a density of 5×10³ cells per well and cultured for 24, 48, 72, and 96 hours, respectively. At each time point, 20 μL of MTT solution (5 mg/mL, purchased from Sigma-Aldrich) was added to each well, followed by incubation at 37°C for 4 hours. After aspirating and discarding the supernatant, 150 μL of dimethyl sulfoxide (DMSO) was added to each well. The plate was then placed on a shaker to ensure complete dissolution of the formazan crystals. Finally, a microplate reader (Bio-Rad Laboratories, Inc.) was used to measure the absorbance at a wavelength of 490 nm, and the cell proliferation rate was calculated with reference to the control group.

***Transwell migration assay***

For cell lines undergoing migration analysis, 2×10⁴ cells resuspended in serum-free medium were added to the upper chamber of a Transwell insert (8 μm pore size, obtained from Corning). The lower chamber was filled with 600 μL of medium containing 10% fetal bovine serum (FBS) to serve as a chemoattractant. After incubation at 37°C for 24 hours, non-migrated cells in the upper chamber were wiped off with a cotton swab. Migrated cells adhering to the membrane of the lower chamber were fixed with 4% paraformaldehyde and then stained with 0.1% crystal violet. Subsequently, the cells were observed under an inverted microscope (Olympus), and cell counts were performed in 5 randomly selected fields of view.

***Transwell invasion assay***

The upper chamber of a Transwell insert (8 μm pore size) was pre-coated with Matrigel (purchased from BD Biosciences) diluted at a ratio of 1:8, followed by incubation at 37°C for 4 hours to allow gel formation. Afterward, 2×10⁴cells cultured in serum-free medium were added to the upper chamber, while 600 μL of medium with 10% FBS was added to the lower chamber. Following 48 hours of incubation at 37°C, non-invaded cells in the upper chamber were removed. Invaded cells that had migrated to the membrane of the lower chamber were fixed, stained, and counted using the same method as described for the Transwell migration assay.

***Mouse xenografts assay***

Male Balb/c nude mice, aged five weeks, were obtained from the Animal Center of the Army Medical University and maintained in accordance with standard specific pathogen-free (SPF) conditions. All animal experimentation adhered to ethical standards for laboratory animal care and use, as sanctioned and monitored by the IACUC of the Army Medical University. A total of 1×10^6^ cancer cells, specifically HT-29, sh-ASCL2/HT-29, SMOC2-OE-sh-ASCL2/HT-29, LS174T, sh-ASCL2/LS174T, and SMOC2-OE-sh-ASCL2/LS174T, were administered subcutaneously into the flank of the mouse in a volume of 100 μl of PBS. This procedure was part of an additional in vivo tumorigenicity assessment. We used 1×10^6^ cancer cells of sh-ASCL2/HT-29, SMOC2-OE-sh-ASCL2/HT-29, sh-ASCL2/LS174T, and SMOC2-OE-sh-ASCL2/LS174T cells to develop mouse xenografts. Five days after SMOC2-OE-sh-ASCL2 cells injection, TGF-β receptors inhibitor (A83-01) ([Beyotime Biotechnology](https://www.so.com/link?m=zNH5R3WaUU1RPeMRneEdHZBg368FsU5oL%2B%2Fe3xGR2Tn6JyMGku9FmTUmtvjdfyNCL3oQJEaKvFGEw%2F9A9ML%2FC%2BCE%2B7hpY8ZASH4w56eHFMAGAfQ5xJpzaOZyGVfuEtWZ20WyTZWKSbrH4PJPGKlneQA%3D%3D) Co, Ltd, Shanghai, China) was injected subperitoneally at 30mg/kg daily into nude mice model five days later after SMOC2-OE-sh-ASCL2 cells inoculation. The tumor sizes were measured and calculated on days 1, 5, 10, 15, 20. The mice were euthanized three weeks after tumor cell inoculation, and the formation of tumors was weighed and divided into two portions: one for RNA analysis, and one for Western blotting assay.

***Luciferase reporter assay***

To confirm whether ASCL2 promotes SMOC2 transcription, a fragment of the SMOC2 5' flanking sequence (spanning the region from -760 to +188 bp) was amplified via PCR techniques, followed by the insertion of the resultant product into the luciferase reporter vector pGL3-Basic. resulting in the construction of the pGL3-Basic-SMOC2 promoter reporter plasmid. Briefly, the SMOC2 promoter sequence was amplified from genomic DNA of Caco-2 cells using primers containing KpnI and HindIII restriction sites. Table 1 presents the primers utilized for the generation of each promoter fragment. Following digestion with KpnI and HindIII, the fragments were effectively inserted into the pGL3-Basic vector. The integrity of all inserted fragments was validated through fluorescein-labeled DNA sequencing.

To investigate the transcriptional regulatory regions within the human SMOC2 promoter, sh-ASCL2/LS174T, sh-ASCL2/HT-29 cell lines, and their corresponding control cells (sh-Ctr/LS174T, sh-Ctr/HT-29) were transfected with three pGL3-based SMOC2 promoter reporter constructs, spanning the regions -760 to +188, -534 to +188, and -176 to +188 respectively. Post-transfection (48 h), luciferase activity was detected using a dual-luciferase reporter assay system. All data are expressed as the mean±standard error (S.E.) from three independent experimental repetitions.

***Chromatin immunoprecipitation*** *(****ChIP) assay***

We conducted ChIP assays utilizing the ChIP kit (Millipore, Catalog Number 17408), following the instructions outlined by the manufacturer. Soluble chromatin was isolated from sh-Ctr/HT-29, sh-ASCL2/HT-29, sh-Ctr/LS174T, and sh-ASCL2/LS174T cell lines. An anti-ASCL2 antibody (MAB4418, Millipore) was used for ChIP. To determine if ASCL2 interacts with the promoter region of SMOC2, primers were designed to amplify specific sequences encompassing binding site 1 (BS1), BS2, BS3, BS4, or BS5 within the human SMOC2 promoter (Figure 2D). The sequences of the primers and the corresponding lengths of the amplicons are detailed in Table 1.

***Bioinformatic analysis***

A retrospective examination was conducted to assess the expression profiles of downstream genes regulated by ASCL2. This analysis utilized volcano plots and heatmaps to compare the gene expression in cells with ASCL2 interference against a control group, employing data sourced from GEO profiles (GSE69036). A correlation analysis was performed to evaluate the relationship between the mRNA expression levels of ASCL2 and SMOC2 in colorectal cancer (CRC) tissues obtained from the GSE13294 dataset. This evaluation employed Spearman's rank correlation test to analyze and compare the mRNA expression levels of ASCL2 and SMOC2 in human CRC specimens with those present in the adjacent peri-cancerous mucosal tissue, as indicated by the GSE62321 dataset. ASCL2, and SMOC2, combined abnormal ASCL2 and SMOC2 expression levels and the clinical information pertaining to 474 colorectal cancer (CRC) samples was sourced from the TCGA database. The optimal cut-off values were determined utilizing X-tile software. The log-rank test was employed to assess its statistical significance. To determine the significantly consistent gene sets between high ASCL2 and SMOC2 mRNAs versus low ASCL2 and SMOC2 mRNAs patient groups, we used the above-mentioned 474 CRC patients and imported their gene expression data into Gene Set Enrichment Analysis (GSEA4.0.1) software to generate enrichment scores for gene sets.

***Statistical analysis***

All experimental procedures were conducted utilizing three distinct biological cell replicates. In the absence of specific indications, the data illustrated in the figures are expressed as the mean± SD. This analysis was derived from the results of three independent trials. The statistical evaluations were performed utilizing GraphPad Prism version 8.0.
