# Supplementary material for: ASCL2-driven SMOC2 expression enables colorectal cancer malignant self-sufficiency via boosting TGF-β1/SMAD3-mediated EMT program
Source: Genes Dis. 2026 Mar 5;14(3):102127. doi: 10.1016/j.gendis.2026.102127 (PMC13718859; doi:10.1016/j.gendis.2026.102127)
Supplement: Supplementary file 2 [file mmc2.docx]

TABLE 1 | The primer sequences used in each **SMOC2** promoter fragment, **qPCR and** ChIP assays

| Type | Sequence | Length of products | Annealing temperature (℃) |
| --- | --- | --- | --- |
| pGL3- SMOC2-promotor  -760/+188 | **5’→3’**  F: 5’-GGGGTACCATGTGTCTGTGAATGATGCC-3’ | 948bp | N |
|  | R: 5’-CCAAGCTTGGTGGCGGAGGTGGCGGGA-3’ |  |  |
| -534/+188 | F: 5’-GGGGTACC GGCAATGTTCTGGGCTGGAC-3’ | 722bp | N |
|  | R: 5’-CCAAGCTTGGTGGCGGAGGTGGCGGGA-3’ |  |  |
| -176/+188 | F: 5’-GGGGTACCCCGCAGACCCGCGCCA-3’ | 364bp | N |
|  | R: 5’-CCAAGCTTGGTGGCGGAGGTGGCGGGA-3’ |  |  |
| -85/+188 | F: 5’-GGGGTACC AGGAGAGGGGGAGGGAGA-3’ | 273bp | N |
|  | R: 5’-CCAAGCTTGGTGGCGGAGGTGGCGGGA-3’ |  |  |
| Primers for qPCR | **5’ →3’** |  |  |
| ASCL2 | F: 5’-CGTGAAGCTGGTGAACTTGG-3’ | 113bp | 63 |
|  | R:5’-GGATGTACTCCACGGCTGAG-3’ |  |  |
| SMOC2 | F: 5’- AAGGAAGTATACCCAGGAGCAA -3’ | 105bp | 60 |
|  | R: 5’- GTGTAGCTGTGACACTGGACCT -3’ |  |  |
| E-CADHERIN | F: 5’-TACACTGCCCAGGAGCCAGA-3’ | 103bp | 60 |
|  | R: 5’-TGGCACCAGTGTCCGGATTA-3’ |  |  |
| N-CADHERIN | F: 5’-GAAGGAGGTGGGGAGGAAGATA-3’ | 131bp | 60 |
|  | R: 5’-GGTGGTCTCTGACGAGGTAAACA-3’ |  |  |
| ZEB1 | F: 5’-CCTGAGTCCTATGTTTCATCAGC-3’ | 128bp | 60 |
|  | R: 5’-AGCTCTTCTGCACTTGGTTGTG-3’ |  |  |
| ZEB2 | F: 5’-ATGGCCTACACCTACCCAAC-3’ | 165bp | 60 |
|  | R: 5’-TTTGCGAGACAGACAGGAGT-3’ |  |  |
| TGF-β1 | F: 5’- CCCTGGACACCAACTATTGC-3’ | 75bp | 60 |
|  | R: 5’- TGCGGAAGTCAATGTACAGC -3’ |  |  |
| SMAD3 | F: 5’- TGGACGCAGGTTCTCCAAAC-3’ | 90bp | 63 |
|  | R: 5’- CCGGCTCGCAGTAGGTAAC -3’ |  |  |
| α-TUBULIN | F: 5’-TTCTGGGTTCACCTCGCTGC -3’ | 133bp | 60 |
|  | R: 3’-GGTGAGGATGGAGTTGTAGGGC -5 |  |  |
| Primers for ChIP | **5’ →3’** |  |  |
| Binding site1 | F: 5’-TGTAAAGATGCCAAAGGCGG -3’ | 102bp | 60 |
|  | R: 5’-CTCAGAGGTGCTGAAGGATCA -3’ |  |  |
| Binding site 2 | F: 5’- GGTGCGTCCCGTGGTT -3’ | 95bp | 60 |
|  | R: 5’-ACACTTACACACACGCACAC -3’ |  |  |
| Binding site 3 | F:’ 5’-GTGTGCGTGTGTGTAAG -3’ | 133bp | 62 |
|  | R: 5’-AGCGAGAGGTGGAGC -3’ |  |  |
| Binding site 4 | F: 5’-AGGGAGGCCTCTGGGC -3’ | 73bp | 60 |
|  | R: 5’-CCAGCCCCACCTCCCA -3’ |  |  |
| Binding site 5 | F: 5’-TATTTGACAGGAGCGAGGGC -3’ | 103bp | 60 |
|  | R: 5’-GATGCTCTCCCCACCGC -3’ |  |  |

N: None
