# Supplementary material for: ASCL2-driven SMOC2 expression enables colorectal cancer malignant self-sufficiency via boosting TGF-β1/SMAD3-mediated EMT program
Source: Genes Dis. 2026 Mar 5;14(3):102127. doi: 10.1016/j.gendis.2026.102127 (PMC13718859; doi:10.1016/j.gendis.2026.102127)
Supplement: Supplementary file 3 [file mmc3.docx]

**TABLE 2 | The antibodies used in the experiment**

| Antibodies | Company | Dilution |
| --- | --- | --- |
| Primary antibodies: |  |  |
| Mouse monoclonal IgG to ASCL2 | Millipore（MAB4418） | WB: 1:1000 |
| Rabbit polyclonal IgG to SMOC2 | Novus(NBP2-20425) | WB: 1:1000 |
| Mouse monoclonal IgG to E-CADHERIN | Abcam (ab1416) | WB: 1:1000 |
| Rabbit polyclonal IgG to N-CADHERIN | Santa Cruz Biotechnology (sc7939) | WB: 1:500 |
| Rabbit polyclonal IgG to ZEB1 | Proteintech (21544-1-AP) | WB: 1:500 |
| Mouse monoclonal IgG to ZEB2 | Santa Cruz Biotechnology (sc271984) | WB: 1:300 |
| Rabbit polyclonal IgG to TGF-β1 | Proteintech (21898-1-AP) | WB: 1:1000 |
| Rabbit monoclonal IgG to p-SMAD3 | Abcam(ab52903) | WB: 1:2000 |
| Mouse monoclonal IgG to SMAD3 | Proteintech (66516-1-Ig) | WB: 1:1000 |
| Rabbit polyclonal IgG to α-TUBULIN | Proteintech (11224-1-AP) | WB: 1:1000 |
| Mouse monoclonal IgG to α-ACTININ | Cell Signaling Technology(#69758) | WB: 1:1000 |
| Secondary antibodies: |  |  |
| HRP-conjugated Affinipure Goat Anti-Rabbit IgG(H+L) | Proteintech （SA00001-2） | WB: 1:10000 |
| HRP-conjugated Affinipure Goat Anti-Mouse IgG(H+L) | Proteintech （SA00001-1） | WB: 1:10000 |
